# Predictors of in-hospital adverse outcomes in aortic surgery

**DOI:** 10.1186/1749-8090-8-S1-O37

**Published:** 2013-09-11

**Authors:** M Sales, C Aguzzoli, A Rösler, E Lúcio, P Leães, V Lima, M Pontes, F Lucchese

**Affiliations:** 1 Cardiovascular Surgery, Hospital São Francisco, Porto Alegre, Brazil

## Background

Aortic surgery is associated with high rates of death and complications. Our aim was to describe short-term outcomes after aortic surgery, and to identify predictors of adverse in-hospital outcomes.

## Methods

All patients operated on for aortic diseases (2009-2012) were included. We evaluated demographic, clinical and operative variables, in-hospital mortality and MACCE.

## Results

We included 235 pts (61±13y, 66%male). Group 1 (aneurysms 61%) and Group 2 (dissections, ulcer, hematoma 36%). Others: 3%. Procedures: aortic root replacement (26,5%), Bentall procedure (23,5%), endovascular (28%), hybrid surgery (19%), aortoplasty and Tirone (3%); concomitant procedures in 20% of cases. Group 2 had higher BP, more urgent and hybrid procedures, greater pump/ischemia time, smaller aortic size and less Bentall procedure. Mortality was 8,5% (Group 1, 4,6%; Group 2, 15,5%, p=0,004). Rate of MACCE was 19,2% (Group 1, 11,3%; Group 2, 33,7%, p<0,001). Reoperation occurred in 7,3%, complications 34,2%, stroke 4,3%, AKIN 7,3%, respiratory complications 15,9%. Medullary ischemia developed in 2 patients (0,8%). By multivariate logistic regression, independent predictors of death were hybrid procedure [OR=7,51 (1,05–53,4) p=0,044], aortic size [OR=1,05 (1,02-1,10) p=0,005] and pump time [OR=1,10 (1,01–1,20) p=0,034]; predictors of MACCE were urgent surgery [OR=7,17 (1,10-49,5) p=0,045], combined aneurysms [22,4 (1,42-353) p=0,027], and concomitant mitral valve surgery [OR=46,5 (1,3-166) p=0,035]. Endovascular procedure was independently associated with reduction of MACCE incidence [OR=0,05 (0,004-0,730) p=0,045].

## Conclusions

Aortic surgery in a specialized center is associated with low rates of in-hospital death and MACCE. Independent predictors of in-hospital death were the hybrid approach, aortic size and bypass time; predictors of MACCE included urgent surgery, combined aneurisms and concomitant mitral surgery. Endovascular approach independently reduced MACCE.

